# Reinforcement generates systematic differences without heterogeneity

**DOI:** 10.1073/pnas.2408163122

**Published:** 2025-06-06

**Authors:** Alexandros Gelastopoulos, Lucas Sage, Arnout van de Rijt

**Affiliations:** ^a^Department of Business and Management, University of Southern Denmark, Odense 05230, Denmark; ^b^Department of Economics and Business, Pompeu Fabra University, Barcelona 08002, Spain; ^c^Barcelona School of Economics, Barcelona 08005, Spain; ^d^Institute for Advanced Study in Toulouse, Toulouse Capitole University, Toulouse 31080, France; ^e^Department of Political and Social Sciences, European University Institute, Fiesole FI 50014, Italy; ^f^Institute of Cognitive Sciences and Technologies, National Research Council of Italy, Rome 00196, Italy

**Keywords:** reinforcement, Pólya urn, cumulative advantage, rich get richer, heterogeneity

## Abstract

Inequality in outcomes may emerge through a reinforcement process in which stochastic variation in values is determined by prior values but may also originate in preexisting differences in unobserved factors. A common approach toward differentiating between these origins in longitudinal data is to attribute systematic differences between units—differences in means or differences proportional to a time-varying group average—to unobserved heterogeneity. We show that any longitudinal data with systematic differences can also be produced by a reinforcement-driven data generating process. This result reconciles findings in three distinct research areas—science of science, personal culture, and sexual networks—where reinforcement is a strong theoretical prior, yet longitudinal data analyses advance an explanation of interpersonal differences based on heterogeneity. Future studies may bound the role of heterogeneity and reinforcement from below by measuring fixed traits that systematically vary with the outcome and isolating random events that trigger emergent differences.

## Significance

In analyses of longitudinal records, it is standard practice to attribute systematic differences across units to heterogeneity in unobserved characteristics. In this paper, we show that such systematic differences might instead be the result of a reinforcement mechanism, where present outcomes are driven by past outcomes. Because reinforcement is not a mere mechanical possibility but also a strong theoretical prior in many research areas, our results suggest that heterogeneity might have been overestimated in previous studies. An explanation that bases interpersonal differences on reinforcement instead of heterogeneity not only changes our understanding of social processes but it can also suggest different targeted interventions for achieving a given policy goal.

Do the rich get richer because they were previously rich or because they are more talented than the poor? If we stretch the meaning of income and talent, then this question has been posed many times in a wide range of scientific disciplines (1–12). The question contrasts two commonly juxtaposed models that both generate temporal sequences of some quantity for a set of units, e.g., individuals. This quantity could in principle be anything, e.g., income, citations, attitude scores. In a heterogeneity model, individuals are each equipped with a certain propensity to generate high or low values, e.g., talent, and interpersonal variability in this propensity is gradually revealed over time through the values that sequentially materialize. In a reinforcement model, individuals ex ante have identical expected values for the focal quantity but some of them initially, by chance, record higher values than others, and positive reinforcement (or, “rich get richer,” “success breeds success,” “the Matthew effect,” “preferential attachment”) leads this initial inequality to persist.

Adjudicating between the two models in longitudinal records is made difficult by the fact that both a reinforcement model and a heterogeneity model, as well as hybrid models, generate data in which some individuals reliably score higher than others (13–19). It is widely known that unaccounted heterogeneity can be misinterpreted for reinforcement or another underlying cause (18, 20–24). Here, we problematize the opposite direction: Data structures that one might naturally think of as stemming from heterogeneity may actually have been generated by reinforcement. More precisely, a common approach toward separating the two origins of variation in longitudinal data is to attribute differences in individuals’ mean scores or differences proportional to a time-varying group average to unobserved heterogeneity (25–32). This presumed unobserved heterogeneity is often represented by fixed or random effects. While it is intuitive to think of such systematic differences as necessitating an explanation in terms of individual-specific traits, what we show in this paper is that any longitudinal data with systematic interpersonal differences can also be generated by a reinforcement-driven data generating process. Because reinforcement is not a mere mechanical possibility but also a strong theoretical prior in many research areas, the practice of interpreting systematic interpersonal differences as grounded in heterogeneity risks misattributing emergent differences to fictive talents or dispositions.

In what follows, we start by illustrating the problem of confounding with a simple example. We next discuss three cases taken from distinct literatures with the following three features:

1. There is a prominent theory that yields a reinforcement hypothesis.
2. A heterogeneity model is fit to the data, which instead interprets interpersonal differences in outcomes as due to outcome-relevant traits that are assumed but not observed.
3. A “twin” reinforcement model can be found that fits the data equally well and attributes all interpersonal differences to reinforcement.

We then generalize from these cases and prove that every heterogeneity model has a reinforcement model twin that generates identical data. Additionally, a hybrid model can always be constructed that fits a given dataset equally well and flexibly partitions interpersonal variability into any desired combination of reinforcement and heterogeneity. We conclude by showing how in practice it is difficult to use parsimony as a criterion for favoring a heterogeneity over a reinforcement interpretation.

## An Illustration: Reinforcement vs. Heterogeneity in Binary Outcomes.

We show how a basic heterogeneity model for binary outcome data is fully confounded with a well-known reinforcement model: the Pólya urn. The Pólya urn can be described as follows: An urn contains one red and one black ball. At each time step, we randomly draw one ball from the urn and then return it together with another ball of the same color. That is, we add one ball at each time step, with the probability of that ball being black proportional to the number of black balls already in the urn. Modifications of this scheme, often allowing many colors and the appearance of new colors, include Simon’s reinforcement model (1), Barabási and Albert’s preferential attachment model (33), and Price’s urn model (5) (see *SI Appendix* for a discussion of these models).

Given that the more black balls we draw the more likely black balls become in the future, this is a reinforcement model. However, it turns out that this model is statistically equivalent to a pure heterogeneity model. Specifically, consider the following alternative model. We first draw a number p uniformly randomly from the interval [0,1]. We then generate a sequence of black and red balls, with the probability of each ball being black equal to p, independently of the colors of the previous balls.^*^

Assuming that p cannot be directly observed, then it is impossible to infer for a given set of sequences (e.g., a panel dataset with repeated binary observations for a sample of individuals) whether a Pólya urn model or the alternative heterogeneity model is more likely to have generated it. This is formalized in the following proposition (34, 35) [see also endnote 1 in ref. 13; for a proof see (36, theorems 2.1 and 2.2) or (37, example 1.5)].

Proposition 1 *Consider a Pólya urn model starting with 1 black and 1 red ball, and let* $Y_{n}$ *denote the outcome of the* n-*th draw with* $Y_{n}=1$ *if a black ball is drawn and* $Y_{n}=0$ *otherwise. Also, let* p *be uniformly distributed on* [0,1] *and let* $Y_{n}^{\prime }$ *be a sequence of binary random variables, conditionally independent given* p, *with* $\mathbb{P}\left(Y_{n}^{\prime }=1|p\right)=p$. *Then, the sequences* $\left\{Y_{1},Y_{2},\ldots \right\}$ *and* $\left\{Y_{1}^{\prime },Y_{2}^{\prime },\ldots \right\}$ *have the same joint probability distribution, i.e., they are statistically indistinguishable*.

**Fig. 1.**
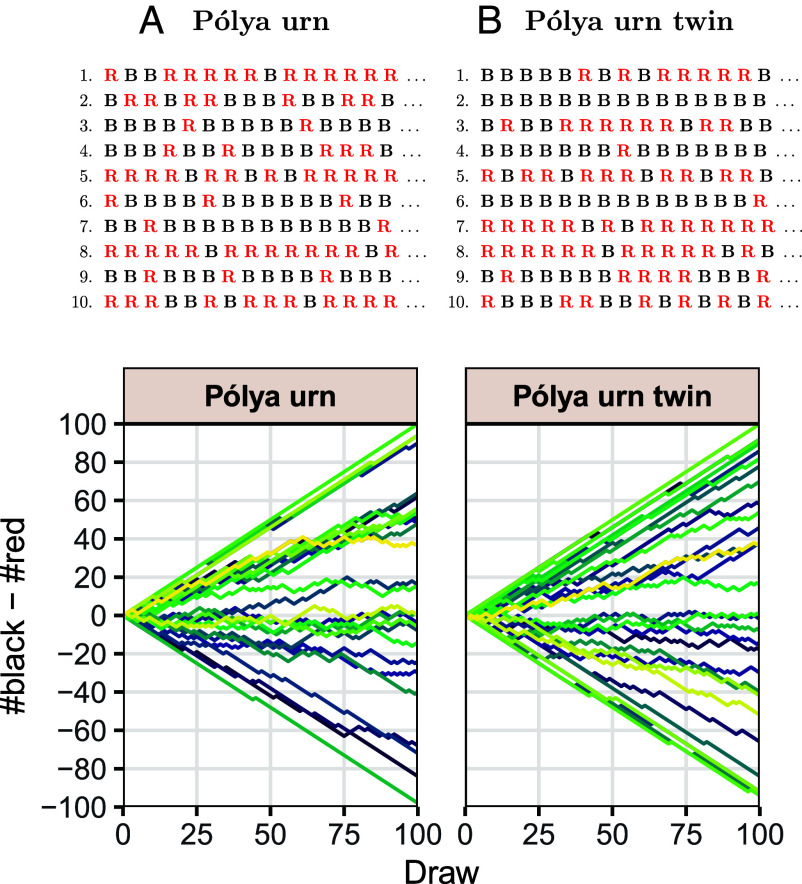
*Top*: 10 simulated sequences. *Bottom*: Difference in the number of black and red balls drawn in 30 simulated sequences. (*A*) Pólya urn model starting with 1 black and 1 red ball. (*B*) A number p is first drawn uniformly from the interval [0,1] and then balls are drawn independently with probability of drawing a black ball equal to p. By *Proposition* 1, the two models are statistically indistinguishable.

See Fig. 1 for an illustration. We emphasize that the proposition pertains to equality of joint probability distributions, i.e., the distributions of entire sequences, which is a much stronger assertion than equality of the distributions of $Y_{n}$ and $Y_{n}^{\prime }$ for each n separately. In terms of Fig. 1, this means that not only the probability of **B** or 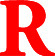 in any given position is the same for the two models, but that the probability of any sequence of draws is exactly the same in the two models. It follows that there is no statistical test that can decide which of the two generative models produced a given set of empirical sequences or even say which one is more likely to have done so.

We now describe cases from the literature where the existence of alternative models that are equally consistent with the data suggest plausible alternative interpretations for the underlying mechanisms.

## Case 1: Cumulative Advantage in Science.

A central thesis in the science of science is that academic achievement is self-reinforcing, known as cumulative advantage (4): Awards lead to more awards, and citations beget further citations. Cumulative advantage would cause luck-based differences in early-career success between two similarly talented academics to perpetuate throughout the career. This is a reinforcement hypothesis (feature 1). Surprisingly, this hypothesis has been repeatedly rejected in analyses of observational data (9) where individual differences in academic success in longitudinal records are found to be entirely due to heterogeneity (26, 30, 38, 39). The fact that analysis of observational data contradicts cumulative advantage is puzzling in light of field-experimental studies supporting it (40–44) as well as the strong theoretical prior of cumulative advantage being “so intuitively plausible that for many it simply must be true” (p. 615 in ref. 45). We resolve this puzzle by arguing that data patterns that resemble heterogeneity plausibly originate in reinforcement instead, or a combination of both. We illustrate this reconciliation using an influential model that was proposed to explain the finding that the best-cited paper occurs randomly in a scientist’s career (30): the Q-model. In this model, each scientist has a “Q-factor,” corresponding to the expected citation impact of a paper they write. This Q-factor is the same for each of a scientist’s papers. Variation in citation impact across papers of the same scientist is exclusively determined by chance. This is therefore a heterogeneity model, denying any role for cumulative advantage in the generation of citation inequalities (feature 2). This model has been found to provide a good fit to longitudinal data on citations across scientists’ careers (30). We show that there exists a reinforcement model that produces identical predictions and therefore must fit the data equally well (feature 3).

In the Q-model, the number of citations $c_{n}$ of the n-th paper of a scientist is the product of their Q-factor, which is fixed throughout the lifetime of the scientist, and a luck component $p_{n}$, with the $p_{n}$’s forming an i.i.d. sequence.^†^ The factor Q and the $p_{n}$’s are assumed to be independent random variables and each is log-normally distributed. As a result, $c_{n}=Q\cdot p_{n}$ is also log-normally distributed. For convenience, we are going to work with the logarithms of these quantities $Y_{n}=\log c_{n}$, T=logQ, and $X_{n}=\log p_{n}$, which are all normally distributed, and for which the relation $Y_{n}=T+X_{n}$ holds. Since the mean of $X_{n}$ can be absorbed into T, we assume that $X_{n}$ has zero mean.

We now introduce an alternative, reinforcement model. Let a∈R and b,c>0 be some parameters, and define $Y_{n}^{\prime }$ recursively so that conditioned on $Y_{1}^{\prime },\ldots ,Y_{n}^{\prime }$, the variable $Y_{n+1}^{\prime }$ is normally distributed, with mean

[1] $$\mu_{n+1}=\frac{c\cdot a+\sum_{k=1}^{n}Y_{k}^{\prime }}{n+c}$$

and variance

[2] $$\sigma_{n+1}^{2}=b\cdot \left(1+\frac{1}{n+c}\right).$$

In particular, for n=0 we get that the unconditional distribution of $Y_{1}^{\prime }$ is normal with $\mu_{1}=a$ and $\sigma_{1}^{2}=b+b/c$.

Note that in the above reinforcement model there are no exogenous differences between individuals. Instead, the value of $Y_{n+1}^{\prime }$ is endogenously determined by the values of $Y_{1}^{\prime },\ldots ,Y_{n}^{\prime }$ and noise. Specifically, $Y_{n+1}^{\prime }$ is normally distributed with known variance and a mean that is a deterministic function of the past performances $Y_{1}^{\prime },\ldots ,Y_{n}^{\prime }$.

The next proposition states that, for matching values of parameters, this generative model is statistically indistinguishable from the Q-model, i.e., any longitudinal data that is consistent with one is equally consistent with the other (proof given in *SI Appendix*).

Proposition 2 *Let* $\sigma_{X}$ *and* $\sigma_{T}$ *be the standard deviation of* $X_{n}$ *and* T *respectively, in the* Q-*model, and let* $\mu_{T}$ *be the mean of* T. *If we take* $a=\mu_{T}$, $b=\sigma_{X}^{2}$, *and* $c=\sigma_{X}^{2}/\sigma_{T}^{2}$, *then the sequences* $\left\{Y_{n}\right\}$ *and* $\left\{Y_{n}^{\prime }\right\}$ *have the same joint distribution*.

**Fig. 2.**
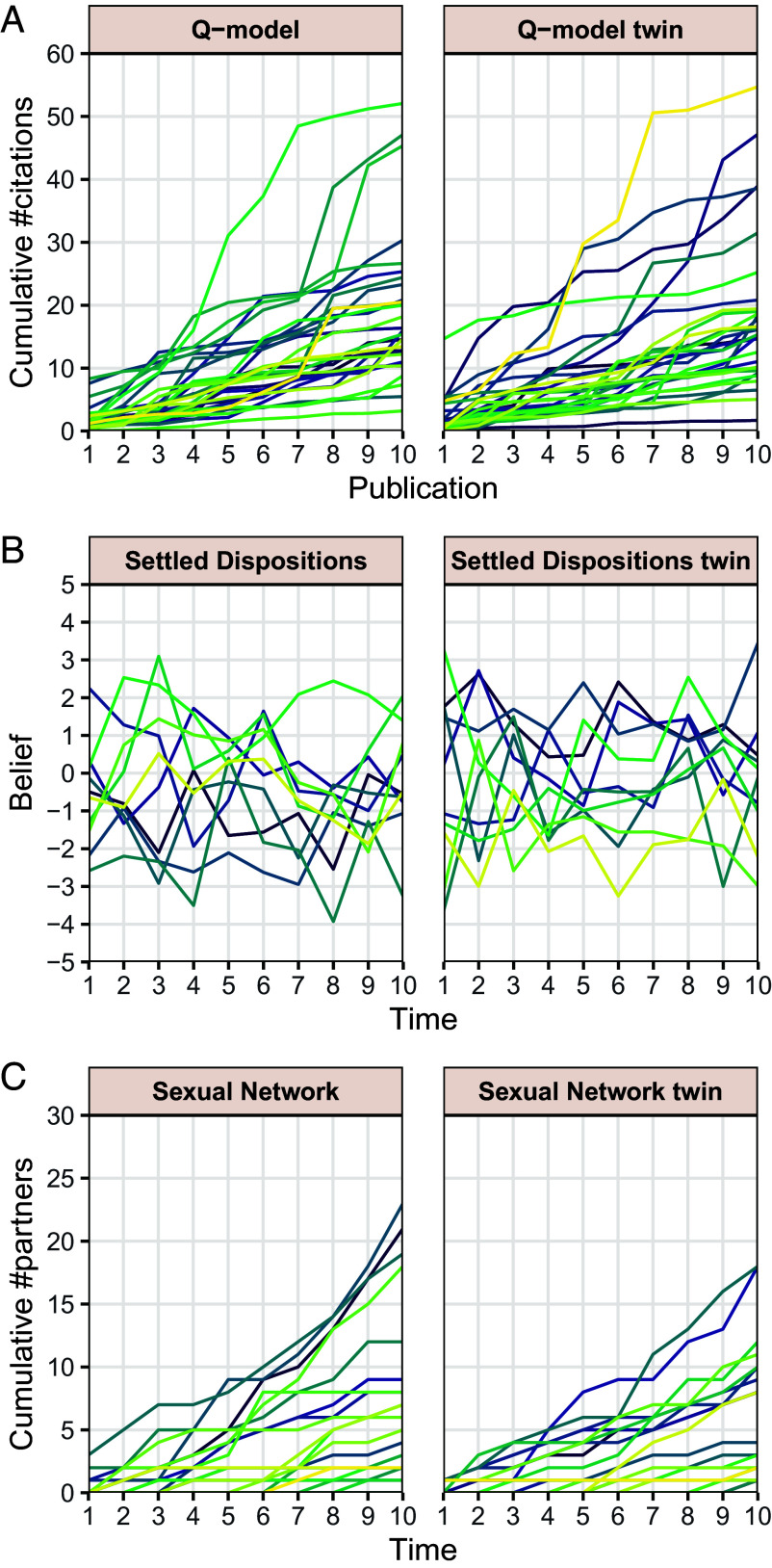
Simulated trajectories of three models and their twins. (*A*) Cumulative numbers of citations across the first ten papers of 30 scientists’ careers in resp. the Q-model (30) with $\mu_{T}=0$, $\sigma_{T}^{2}=0.2$, and $\sigma_{X}^{2}=1$ (*Left*) and its pure reinforcement twin (*Right*). (*B*) Beliefs predicted to be held by 10 individuals in panel data by resp. the Settled Dispositions Model (31) (*Left*) and its pure reinforcement twin (*Right*). (*C*) Cumulative number of partners of 30 individuals in resp. the Sexual Network Model (29) with α=1, $\pi_{0}=0.25$, and $\pi_{j}=0.25\cdot j^{0.5}$ for j≥1 (*Left*) and its pure reinforcement twin (*Right*).

**Fig. 3.**
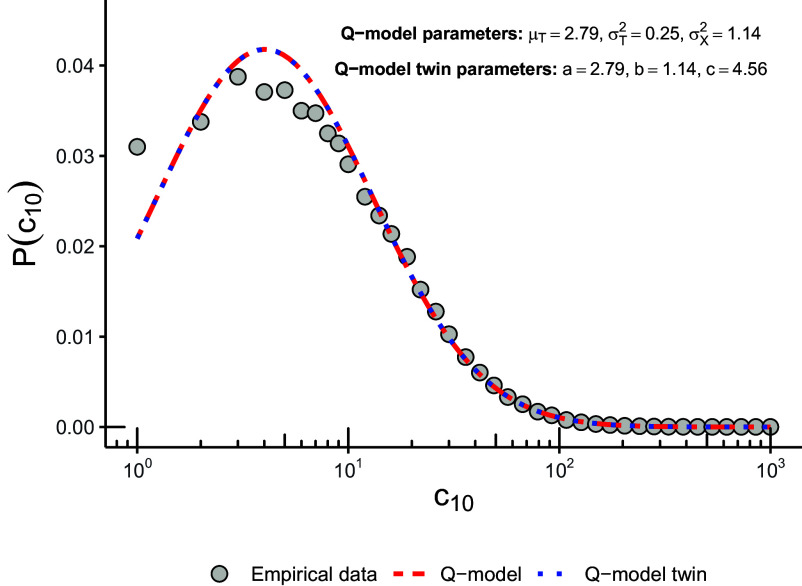
Distribution of the number of citations accumulated 10 y after publication ($c_{\text{10}}$) of articles in APS journals until 2009, following ref. 30. Sample restricted to scientists with at least 50 publications (N = 2,908) and to their 50 first publications. A lognormal distribution with $\mu_{\;\text{empi}}=2.79$ and $\sigma_{\;\text{empi}}=1.18$ was fit using maximum likelihood. The two curves correspond to predictions from the Q-model with $\mu_{T}=\mu_{\;\text{empi}}$ and $\sigma_{X}^{2}+\sigma_{T}^{2}=\sigma_{\;\text{empi}}^{2}$ and its twin with $a=\mu_{\;\text{empi}}$, $b=\sigma_{X}^{2}$ and $c=\sigma_{X}^{2}/\sigma_{T}^{2}$.

Like *Proposition* 1, the assertion of this proposition (and all theorems and propositions below) concerns equality of distributions of entire sequences, not of each term separately. Consequently, no statistical test can distinguish the two models. Panel (*A*) in Fig. 2 shows simulated trajectories of the two models and Fig. 3 their equally good empirical fit to the data analyzed in ref. 30. Equality of joint distributions implies in particular that any cross-time covariances or other moments will be equal for the two processes. Additionally, goodness-of-fit measures will by definition conclude that the two models fit any data equally well, so they cannot be used to favor one model over the other.

### Discussion.

The Q-model twin, like other reinforcement models, such as the Pólya urn model (37) and the standard preferential attachment model (33), has the property that the expected future value of the outcome grows proportionally to the average of past values. It follows that two individuals who are ex ante equivalent, one of which incidentally experiences a success that the other does not, can from that moment on expect to experience success trajectories that are proportional to one another. This predicted pattern is approximately observed in quasi-experimental studies on cumulative advantage: Van de Rijt et al. (46) find that arbitrary successes bestowed upon recipients in four different contexts increase success rates by a multiplier that is mostly stable over time. Bol et al. (42) find that winners in a European grant competition with evaluation scores falling just above the funding threshold bring in euros in subsequent competitions at a rate that hovers around what is roughly twice the rate of those who scored just below the funding threshold. Chan et al. (47) find that publication and citation success of John Bates Clark award winners and nonwinners diverge in a gradual, linear fashion. The proportionality of feedback is also consistent with observational data showing approximately linearly diverging cumulative publication counts for women and men and the hybrid “limited differences model” combining heterogeneity with reinforcement to account for the data (48).

Existing causal evidence on cumulative advantage in science as well as systematic differences in academic success by observable traits (e.g., class, gender, ethnicity) indicate that both reinforcement and heterogeneity play at least a minimal role. The result established here implies that longitudinal records of success fitting a pure heterogeneity model does not teach us that heterogeneity is the primary mechanism. The empirically documented random timing of a scientist’s highest-impact paper does not imply a world in which personal characteristics (e.g., ability) are the main source of systematic distinction and where cumulative advantage plays a negligible role. It equally permits the interpretation of a world driven by cumulative advantage, wherein comparable scientists experience dramatically different careers as a result of chance-based advantages that are perpetually reinforced.

## Case 2: Personal Culture.

There exist two competing models of how the attitudes and preferences that individuals hold change over the course of their lives (31, 32). The Active Updating Model (AUM) has individuals modify their stances in response to new and changing experiences, discourses, and environments (49–51). The AUM is a reinforcement model (feature 1): The process of updating posits that present attitudes are based on previously held attitudes, adjusted to new experiences. The Settled Dispositions Model (SDM) instead asserts that individuals have preformed beliefs, attitudes, and preferences (formed early in life, for example) that remain stable over the life course (52, 53). Although the expression of these beliefs may fluctuate from one period to the next, the underlying beliefs remain the same throughout adult life. The SDM is a heterogeneity model. It has been found that most variability across hundreds of attitude items in large-scale longitudinal data is due to either between-individual differences in baseline personal culture or unsystematic fluctuations around that baseline, consistent with the SDM or a mixed model that involves little permanent change (31, 32) (feature 2). The minor systematic change in personal culture observed in past studies limits the role for causes of systematic change theorized and evidenced elsewhere (32): Personal development is thought to occur throughout the life course (54), with people changing attitudes in response to changing social environments, technological progress, political events, or under normative pressure (55–57).

The AUM has previously been formalized as a first-order Markov process, in which only attitudes held in the recent past inform current attitudes (31, 32). Life course theories however conceptualize the life course instead as a self-referential biographical process (32), in which individuals’ actions are informed by experiences in both the distant and more recent past (58–60). We propose that, in line with this conceptualization of the life course, variance attributed to settled dispositions could instead plausibly represent systematic belief change. Specifically, we show that the SDM is statistically equivalent to a reinforcement model that is an alternative formalization of the AUM (feature 3). It follows that beliefs that fluctuate around individual-specific averages can equally be accounted for by a theory of active updating in which change is systematic.

In the SDM, the expression of the belief at period n is given by $y_{n}=U+v_{n}$, where U is the underlying belief, fixed for the individual, and $v_{n}$ is a random term that captures the individual’s recent experience. This equation is formally equivalent to the Q-model discussed in the previous section, with U replacing T (=logQ) and $v_{n}$ replacing $X_{n}$ ($=\log p_{n}$). For simplicity, let us assume that both U and $v_{n}$ are normally distributed with zero mean and unit variance, so that the resulting model is exactly equivalent to the Q-model with the extra assumptions $\sigma_{T}^{2}=\sigma_{X}^{2}=1$ and $\mu_{T}=0$. Then, *Proposition* 2 shows that this model is statistically equivalent to the reinforcement model

[3] $$y_{n}^{\prime }=\frac{n-1}{n}\cdot {\overset{¯}{y}}_{n-1}^{\prime }+\sqrt{1+\frac{1}{n}}\cdot v_{n},$$

where ${\overset{¯}{y}}_{n-1}^{\prime }$ is the average of $y_{1}^{\prime },\ldots ,y_{n-1}^{\prime }$. See Fig. 2*B*.

### Discussion.

Conceptually, the reinforcement model we identify takes a middle position between the (Markovian) AUM in which only the recent past informs the present and the SDM in which only the distant past informs the present. One aspect of the particular form of updating in the twin reinforcement model we have identified is that noise diminishes in magnitude over time, as reflected in the decreasing coefficient on $v_{n}$ in Eq. **3**. Such a decrease is consistent with the typical life course in modern societies in which as individuals age they are less exposed to transformative events and changing environments (32, 54, 61). The key implication of these results is that longitudinal evidence previously found to favor the settled dispositions theory over the theory of active updating, can also be taken to support the latter theory.

## Case 3: Preferential Attachment in Sexual Networks.

Sexual networks approximate a scale-free degree distribution characterized by a small number of individuals with many sexual partners (62). Such individuals likely play a disproportionate role in the spreading of sexually transmitted diseases (STDs). Determining the causes of high sexual activity aids targeted public health interventions with the identification of select individuals who will likely have many future sexual partners. Theoretical arguments for preferential attachment (feature 1) in the context of sexual networks include the greater perceived attractiveness of individuals known to have had many sexual partners, the habit and addictiveness of regularly changing sexual partners, experience-driven romantic and sexual ability, and the reinforcement of sexually liberal norms (29). Models in this literature partition longitudinal variation in sexual contacts of individuals into a heterogeneity and a reinforcement component (feature 2). In the Sexual Network Model (SNM) in ref. 29, there is a person-specific factor κ, drawn from a $Gamma\left(\alpha ,1/\alpha \right)$ distribution,^‡^ which remains fixed over the lifetime of the individual. Conditioned on the value of κ, the number of sexual contacts of the individual is described by a pure-birth process $\left\{Y_{t}\right\}$,^§^ with the instantaneous rate of transitioning from state $Y_{t}=j$ to j+1 equal to $\kappa \cdot \pi_{j}$, where $\pi_{0},\pi_{1}\ldots$ are some fixed positive real numbers.^¶^ Thus, this model incorporates both heterogeneity, through the variable κ, and reinforcement, through the state-dependent factor $\pi_{j}$. Applied to longitudinal data on sexual activity, the model finds an important role for heterogeneity, while it finds reinforcement to have a sublinear form, so that reinforcement alone cannot account for the extreme variability in data on sexual partnering.

We now introduce a pure reinforcement equivalent of this model (feature 3). Define $\left\{Y_{t}^{\prime }\right\}$ to be a regular point process (63), such that conditioned on $Y_{t}^{\prime }=m$ and on the times of the first m events, $t_{1},\ldots ,t_{m}$, the instantaneous rate at time t is^#^

[4] $$\lambda_{m+1}\left(t|t_{1},\ldots ,t_{m}\right)=\frac{\pi_{m}\cdot (m+\alpha )}{\pi_{m}\cdot t-\sum_{j=1}^{m}\left(\pi_{j}-\pi_{j-1}\right)t_{j}+\alpha },$$

where the parameter α and the $\pi_{j}$’s take the same values as in the SNM. Observe that this definition contains no notion of heterogeneity; the rate of new sexual contacts is completely determined by the history of the process, i.e., it is a pure reinforcement model. Another difference from the SNM is that the rate of new sexual contacts is determined not only by the number of previous contacts but also by when they occurred; the more recent the contacts, the higher the instantaneous rate. The following proposition asserts that the two models are statistically indistinguishable in longitudinal data.

Proposition 3 *The processes* $\left\{Y_{t}\right\}$ *and* $\left\{Y_{t}^{\prime }\right\}$ *have the same joint distribution*.

The proof is given in *SI Appendix*. Fig. 2*C* shows simulations of the two models.

### Discussion.

The public health challenge informed by these results is the allocation of resources for STD prevention. The substantial role for heterogeneity in the SNM would seem to imply that to a significant degree individuals with high numbers of future sexual partners can be identified a priori on the basis of intrinsic properties, when these are observable, and then targeted by public health campaigns. The pure reinforcement model we identified that fits the data equally well instead suggests that the only relevant data for predicting numbers of future sexual partners is the personal history of sexual partnering realized so far. Such a reinforcement-based reading of the results suggests a very different policy, namely one of targeting actual hubs in sexual networks (64) rather than identifying categories of risky people. Any mix between the two models is statistically equally viable too. The specific split between heterogeneity and reinforcement that the SNM finds depends on the specific parametric form used for the $\pi_{j}$’s and crucially on the assumption that the timing of previous sexual contacts does not matter. When assuming a different parametric form for the $\pi_{j}$’s or some form of time dependence, a different amount of heterogeneity is found. While effective differentiation between heterogeneity and reinforcement may therefore be difficult, a lower bound on the role of heterogeneity can be established by measuring stable traits that systematically vary with the outcome, which can then be used in targeted interventions. For example, men report higher numbers of sexual partners than women (29), suggesting that interpersonal variation cannot be due to reinforcement alone.

## Generalization

The three cases we have presented demonstrate that in a variety of settings and for various types of longitudinal data, outcome differences that seem to stem from unobserved inherent characteristics of individuals might in fact be the result of a reinforcement process. This raises the question of how general this phenomenon is. In this section, we begin by briefly reviewing other cases where systematic interpersonal differences are attributed to heterogeneity, and then we provide general mathematical results regarding the existence of reinforcement models.

Attributing systematic interpersonal differences in outcomes to heterogeneity is a widespread practice in the empirical and statistical literature. By systematic differences, we mean either differences in means or differences proportional to a possibly time-varying group average. For example, time-constant productivity among creative professionals has been taken as evidence against cumulative advantage and gamma distributed differences in rates of productivity as evidence for differences in giftedness (38, 39, 65–68). Longitudinal stability of cross-sectional variation has been used as evidence against cumulative advantage (26). In the conditional frailty model (28), the multiplicative heterogeneity-dependent factor can absorb all interindividual differences that remain constant in proportion over time. Similarly, in the proportional hazards model (69), the heterogeneity factor in the expression for the hazard rate captures all variation that remains proportionally constant over time. The constancy of event rates over time has been proposed as a test that distinguishes between heterogeneity and contagion for models of event statistics (27). And in life-cycle earnings modeling that aims to distinguish heterogeneous growth rates from variation due to shocks with permanent effects (19, 70–74), heterogeneity is modeled as a linear fixed or random effects term. We therefore ask, under what circumstances can we mistake reinforcement for heterogeneity in imagined characteristics?

In order to answer this question, we need general definitions of heterogeneity and reinforcement. Let us define a (pure) heterogeneity model as a procedure that generates a sequence of observations $\left\{Y_{n}\right\}$, typically taking values in some subset of R or $R^{d}$,^‖^ whose n-th term is a function of i) a latent variable U and ii) a chance component $X_{n}$, with the sequence $\left\{X_{n}\right\}$ consisting of i.i.d. random variables. In order to account for multiple individuals, we will also assume that U is a random variable that is drawn from a fixed distribution. In principle, we make no assumptions about the form of the dependence of $Y_{n}$ on U and $X_{n}$; that is, we let the n-th observation be given by $Y_{n}=f_{n}\left(U,X_{n}\right)$, where the $f_{n}$’s are any measurable functions.^**^ A special case is when $f_{n}\equiv f$ for all n, in which case we get a stationary heterogeneity model. Cases 1 and 2 above involve stationary heterogeneity models. For example, in the case of the Q-model of citations, we had $f\left(U,X_{n}\right)=U+X_{n}$.

Next we describe what we mean by a reinforcement model. A (pure) reinforcement model is one in which there is no latent variable, but instead the n-th observation $Y_{n}$ depends on a combination of luck (modeled as an i.i.d. sequence $\left\{X_{n}\right\}$, as before) and the values of the previous observations, $Y_{1},\ldots ,Y_{n-1}$. Again, we make no assumptions about the form of these dependencies. That is,

[5] $$Y_{n}=g_{n}\left(Y_{1},\ldots ,Y_{n-1},X_{n}\right),$$

where $g_{n}$ can be any measurable function. For example, for the Pólya urn one could define

[6] $$g_{n}\left(Y_{1},\ldots ,Y_{n-1},X_{n}\right)=\left\{\begin{array}{cc} 1, & \;\text{if}X_{n}<\frac{1+\sum_{k=1}^{n-1}Y_{k}}{n+1},\\ 0, & \;\text{otherwise}, \end{array}\right.$$

with the $X_{n}$’s being i.i.d. uniformly distributed on [0,1] (see *SI Appendix* for a derivation).

Finally, by a mixed model we mean one in which $Y_{n}$ may depend on both previous observations and a latent variable U, i.e., $Y_{n}=h_{n}\left(U,Y_{1},\ldots ,Y_{n-1},X_{n}\right)$. The Sexual Network Model (Case 3) is a mixed model, but in continuous time.

We note that while $\left\{Y_{n}\right\}$ will typically represent the sequence of outcomes for a single individual, in principle it can also represent the outcomes of a group of individuals, for example by taking $Y_{n}$ to be a vector variable, and Eq. **5** can express dependencies of one’s records on the past outcomes of others. In *SI Appendix*, we show how several well-known reinforcement models, including preferential attachment models, multiplicative processes (i.e., processes that obey Gibrat’s law), and Price’s urn model can be expressed in terms of Eq. **5**.

We begin by formalizing the well-known fact that allowing for unobserved heterogeneity with unrestricted effects on the data can account for any pattern.

Theorem 4 *Let* $\left\{Y_{n}\right\}$ *be any stochastic process. Then, there exists a heterogeneity model generating a sequence* $\left\{Y_{n}^{\prime }\right\}$ *with the same joint distribution as* $\left\{Y_{n}\right\}$.

**Proof** Let U be uniformly distributed on [0,1]. By the randomization lemma (75, lemma 3.22), there exists some measurable function $f:U\mapsto (Y_{1}^{\prime },Y_{2}^{\prime },\ldots )$ such that $\left\{Y_{n}^{\prime }\right\}$ has the same distribution as $\left\{Y_{n}\right\}$. Denoting the n-th component of f by $f_{n}$, we have that $Y_{n}^{\prime }=f_{n}\left(U\right)$.

Here, the original stochastic process $\left\{Y_{n}\right\}$ can be thought of as representing the empirical process, i.e., the longitudinal data. Specifically, the observations over time for each individual/unit of observation produces one “trajectory” of this stochastic process. By looking at a large number of such trajectories, we can reconstruct the probability distribution over all possible trajectories, i.e., the distribution of $\left\{Y_{n}\right\}$. The theorem says that for a given distribution of trajectories, we can always come up with a pure heterogeneity model whose theoretical distribution (over longitudinal trajectories) coincides with the empirically observed distribution.^††^

As a corollary, we get that any reinforcement or mixed model has a pure heterogeneity model twin.

Corollary 5 *For any reinforcement or mixed model that generates a sequence* $\left\{Y_{n}\right\}$, *there exists a heterogeneity model generating a sequence* $\left\{Y_{n}^{\prime }\right\}$ *that has the same joint distribution as* $\left\{Y_{n}\right\}$.

Our central result is in the opposite direction: Any longitudinal data can also be generated by a pure reinforcement model.

Theorem 6 *Let* $\left\{Y_{n}\right\}$ *be any stochastic process. Then, there exists a pure reinforcement model generating a sequence* $\left\{Y_{n}^{\prime }\right\}$ *with the same joint distribution as* $\left\{Y_{n}\right\}$.

**Proof** Let $\left\{X_{n}\right\}$ be i.i.d. random variables, uniformly distributed on [0,1]. We define $g_{n}$ inductively as follows. Suppose that $g_{1},\ldots ,g_{n-1}$ have already been defined such that $\left(Y_{1}^{\prime },\ldots ,Y_{n-1}^{\prime }\right)$ has the same distribution as $\left(Y_{1},\ldots ,Y_{n-1}\right)$, where $Y_{k}^{\prime }=g_{k}\left(Y_{1}^{\prime },\ldots ,Y_{k-1}^{\prime },X_{k}\right)$ for k∈{1,…,n−1}. By the transfer theorem (75, theorem 6.10), there exists some measurable function $g_{n}$, such that even $\left(Y_{1}^{\prime },\ldots ,Y_{n}^{\prime }\right)$ has the same distribution as $\left(Y_{1},\ldots ,Y_{n}\right)$, where $Y_{n}^{\prime }=g_{n}\left(Y_{1}^{\prime },\ldots ,Y_{n-1}^{\prime },X_{n}\right)$. It follows inductively that all finite-dimensional joint distributions of the two processes $\left\{Y_{n}\right\}$ and $\left\{Y_{n}^{\prime }\right\}$ are equal. By the Kolmogorov extension theorem, the entire sequences $\left\{Y_{n}\right\}$ and $\left\{Y_{n}^{\prime }\right\}$ also have equal joint distributions.

As a corollary, every heterogeneity or mixed model has a pure reinforcement twin that makes the same predictions.

Corollary 7 *For any heterogeneity or mixed model that generates a sequence* $\left\{Y_{n}\right\}$, *there is a pure reinforcement model generating a sequence* $\left\{Y_{n}^{\prime }\right\}$ *with the same joint distribution as* $\left\{Y_{n}\right\}$.

The above theorems and corollaries together imply that by looking at longitudinal data alone, it is in principle impossible to tell whether the variation is due to heterogeneity or reinforcement. One can also get an intermediate model with any desired degree of reliance on reinforcement and heterogeneity that fits the data equally well. One way to do this is to first obtain the two pure models, which we know exist by *Theorems* 4 and 6, and then consider the mixed model in which a sequence is generated from the first model with probability q and from the second with probability 1−q.

## Model Complexity

When two models make the same predictions, it is natural to try to favor one over the other on the basis of simplicity or parsimony. For example, the twin of the Q-model requires that the mean of the n-th observation gives equal weight to all past observations (Eq. **1**) as well as that the variance of the noise component in Eq. **2** is decreasing with time at a certain rate. In contrast, the Q-model has a simpler description without any artificially introduced rate or symmetry. Note that by being statistically equivalent to its twin, the Q-model does make corresponding predictions, namely that conditionally on the past, the mean of the next observation is a function of the average of previous observations, while its variance (conditionally on the past) is decreasing with time at the specified rate; but in the case of the Q-model these are natural consequences of the fact that the uncertainty about one’s talent diminishes as the citation impact of past publications materializes, and each previous publication is equally informative about the talent. Thus, given that in the twin model these properties seem artificially introduced, one might be inclined to favor the Q-model as having a more natural or simpler form, despite having the same number of parameters. In other cases, similar arguments might be possible in the opposite direction. Here, we consider in detail the case of stationary heterogeneity models and their twins, for which the above simplicity argument made for the Q-model can be generalized. We claim that such an argument cannot be maintained unless the predictions corresponding to a property that appears natural in one model and artificial in the other are confirmed by the data. We then show that in various cases we consider, the simplicity property is instead rejected in the data.

### The Simplicity of Stationary Heterogeneity Models.

Stationary heterogeneity models have a particularly simple form, because each observation is generated in the same way (no dependence on n) and independently of past observations conditionally on the heterogeneity variable U. However, the observations are not (unconditionally) independent, because they are linked through the value of U. Specifically, each additional observation reduces our uncertainty about U, which allows us to make better predictions about the next observation. As a result, the variance of $Y_{n}$ when conditioned on the entire past $Y_{1},\ldots ,Y_{n-1}$, decreases for larger n. Moreover, because any past observation is equally informative about U, the conditional distribution of $Y_{n}$ is necessarily symmetric with respect to all past observations. These properties, which arise naturally for stationary heterogeneity models, have to be built into a reinforcement model. For example, the decreasing conditional variance often takes the form of decreasing noise for larger n (see the decreasing noise coefficient in Eqs. **2** and **3**). Even when such decreasing noise might be justified, for example because people’s environment becomes more predictable as they age, the reinforcement model requires a very specific rate for the noise to decrease, which is quite restrictive. As a result, one might conclude that stationary heterogeneity models should generally be favored over their twins on the basis of making fewer or simpler assumptions.

Importantly, however, the above argument applies only when stationarity is a property of the empirical data, not just of the model. By suggesting a stationary heterogeneity model, the researcher imposes this property which is often unnatural to reproduce in an exact way with a reinforcement model (although not always; see the Pólya urn example). But if we allow that stationarity holds only up to a rough approximation in the empirical data, then there is no requirement of the reinforcement model to have the exact quantitative form that is suggested. As an example, the variance in Eq. **2** of the Q-model twin could be considered as simply one possible form from a family of models that make approximately the same predictions, some of which do not require that this variance decreases monotonically with n. Similarly, the dependence of both the Q-model twin and the SDM twin on an equally weighted average of all past observations (Eqs. **1** and **3**) can be seen as just one instance of a more general qualitative statement that there is dependence on the past, rather than a requirement that the dependence takes this particular form.

Consequently, if we want to argue that a stationary heterogeneity model should be favored over its reinforcement twin, we need to check that stationarity is a property of the data, rather than one that the model imposes. But so far, we have defined stationarity in terms of the generative model. In what follows, we turn to the question of how this can be checked in data.

### Stationary Heterogeneity Models Produce Exchangeable Data.

Recall that stationary heterogeneity models are those that can be written in the form

[7] $$Y_{n}=f\left(U,X_{n}\right),$$

i.e., where $f_{n}\equiv f$ does not depend on n, and recall that the $X_{n}$’s are i.i.d. From Eq. **7**, it is evident that stationarity puts some major restrictions on the types of data that can be generated. For example, given that the $X_{n}$’s are i.i.d., the cross-sectional statistics (i.e., the distribution of each $Y_{n}$ separately) must remain necessarily constant with n. Thus, if we find empirically, for example, that $E_{\left[Y_{n}\right]}\neq E_{\left[Y_{m}\right]}$ for some m≠n, then it is impossible for a stationary heterogeneity model to account for these data. But constant cross-sectional statistics are not enough. The necessary and sufficient condition for a set of longitudinal data to be consistent with a stationary heterogeneity model is that they have the exchangeability property.

Exchangeability means that the statistics of the data remain unchanged when the order of observations is shuffled. More precisely, the sequence $Y_{1},Y_{2},\ldots$ is exchangeable if it is statistically indistinguishable from $Y_{\sigma_{1}},Y_{\sigma_{2}},\ldots$ for any finite permutation $\sigma_{1},\sigma_{2},\ldots$ of the positive integers.^‡‡^ Every i.i.d. sequence is exchangeable, but an exchangeable sequence need not consist of independent random variables in general. An example of an exchangeable process that is not i.i.d. is the sequence of draws from a Pólya urn (37, section 1.7). De Finetti’s theorem (34) asserts that a sequence of random variables is exchangeable if and only if it can be written as a mixture of i.i.d. sequences, i.e., a sequence of variables which are conditionally i.i.d. given some parameter, but with this parameter being a random variable itself. This is essentially what a stationary heterogeneity model is. We therefore have the following theorem:

Theorem 8 *Let* $\left\{Y_{n}\right\}$ *be any stochastic process. Then, the following two statements are equivalent*:

1. *There exists a stationary heterogeneity model* $Y_{n}^{\prime }=f\left(U,X_{n}\right)$ *such that* $\left\{Y_{n}^{\prime }\right\}$ *has the same joint distribution as* $\left\{Y_{n}\right\}$.
2. $\left\{Y_{n}\right\}$ *is exchangeable*.

**Proof** For any stationary heterogeneity model $Y_{n}^{\prime }=f\left(U,X_{n}\right)$, conditioned on U, the sequence $\left\{Y_{n}^{\prime }\right\}$ is i.i.d., because $\left\{X_{n}\right\}$ is. Therefore, $\left\{Y_{n}^{\prime }\right\}$ is a mixture of i.i.d. sequences, with mixing distribution equal to that of U. By the converse of De Finetti’s theorem, $\left\{Y_{n}^{\prime }\right\}$ is exchangeable.

For the converse, suppose that $\left\{Y_{n}\right\}$ is exchangeable. By De Finetti’s theorem, it is a mixture of i.i.d. sequences, i.e., there is some random variable U such that conditioned on U, the $Y_{n}$’s are i.i.d. Let $\left\{X_{n}\right\}$ be i.i.d uniformly distributed on [0,1] and independent of U. By the transfer theorem (75, proposition 6.10), there exists some measurable function f such that $f(U,X_{1})$ has the same distribution as $Y_{1}$. Define $Y_{n}^{\prime }=f\left(U,X_{n}\right)$. Since $\left\{X_{n}\right\}$ is i.i.d. and independent of U, we get that $Y_{n}^{\prime }=f\left(U,X_{n}\right)∼f\left(U,X_{1}\right)∼Y_{1}∼Y_{n}$, where ∼ stands for equality of distributions. Therefore, conditioned on U, both sequences $\left\{Y_{n}\right\}$ and $\left\{Y_{n}^{\prime }\right\}$ are i.i.d. and with the same distribution. By integrating over U, we get that $\left\{Y_{n}\right\}∼\left\{Y_{n}^{\prime }\right\}$ even unconditionally.

### Testing Data for Exchangeability.

We now return to Case 1 (cumulative advantage) and Case 2 (personal culture) that both involved stationary heterogeneity models, so that heterogeneity could be favored over reinforcement on parsimony grounds. For both cases, we check whether the exchangeability property holds and that the available data thus support the assumption of stationarity. To test for exchangeability in data, one can conduct any randomness test. We conducted a runs test in each case.

**Fig. 4.**
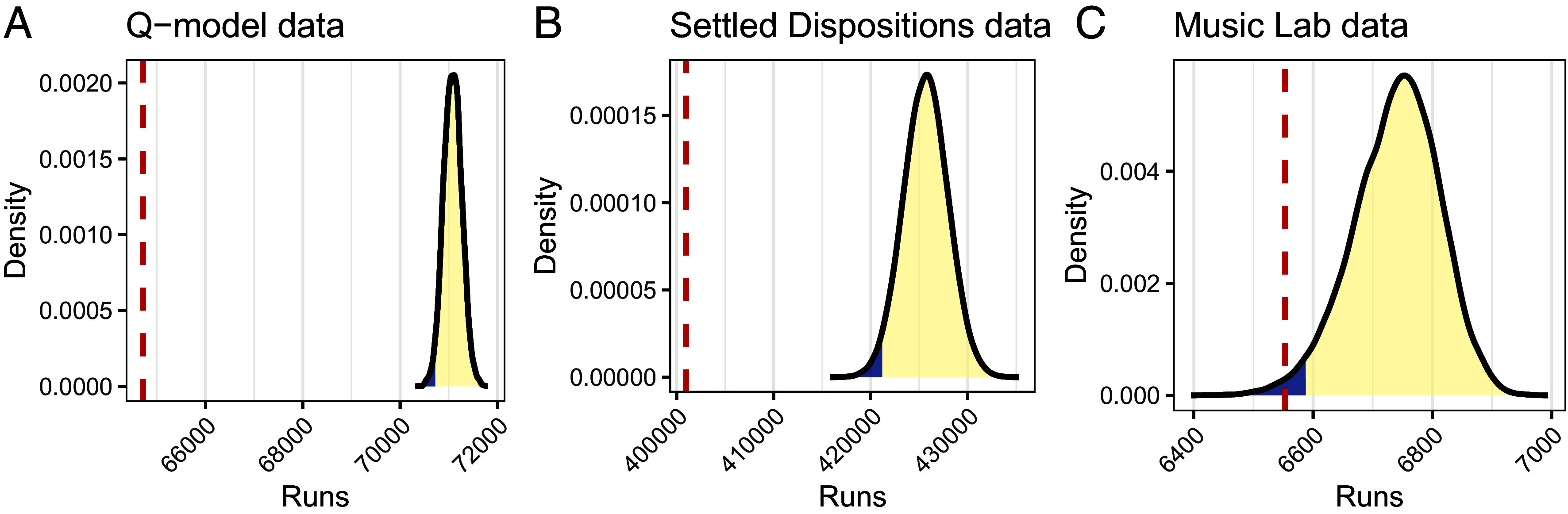
Runs tests of exchangeability. Observed data (dashed red line) and 10,000 permutations (yellow distribution with 2.5% lowest values in blue). (*A*) Citations data to which the Q-model was fit (30). (*B*) Attitudes data to which the SDM was fit (32). (*C*) Song download data from the control condition of the Music Lab experiment (78).

We tested for exchangeability in the data that were used to support the Q-model (30). We did so by dichotomizing the citation data as falling below or above an individual scientist’s median number of citations. We then summed the number of runs—sequences of consecutive zeros or ones—across all scientists. We also calculated this sum of runs for 10,000 permuted datasets in which each scientist’s records were randomly reordered. Panel (*A*) of Fig. 4 shows that the sum of runs in the unpermuted data is much lower than in each of the permuted datasets, showing statistical significance at least at the $2\cdot 10^{-4}$ level.^§§^ In *SI Appendix*, we further show that the specific prediction of the models that the conditional variance of $Y_{n+1}$ given the history decreases at the specified rate (Eq. **2**) is not satisfied, eliminating specifically the argument that the heterogeneity model should be favored because it naturally accounts for this rate. Finally, the most highly cited papers of a researcher have been found more likely than chance to appear close together (76), showing that symmetric correlation of $Y_{n+1}$ with all past observations does not hold in practice either, hence the fact that symmetry is natural to stationary heterogeneity models cannot be used to favor the original model either. All in all, we conclude that the fact that the original model more easily accounts for specific properties cannot be used to favor it, because these properties are not properties of the data; instead, they merely follow from the form of the original model, which was likely selected for simplicity (*Discussion*). We also note that the failure of exchangeability does not show that a heterogeneity mechanism is inconsistent with the data, since a nonstationary heterogeneity model does not have to produce exchangeable data.

To test exchangeability in the data on personal culture, we used the Panel Study of Income Dynamics (77) (see *SI Appendix*, *Extended Methods* and ref. 32 for a description), excluding attitude items that appeared in fewer than three waves of the study, which is the minimum number of observations required in order for the number of runs to be affected by a shuffling of the indices. For each item, we excluded participants who responded in fewer than three waves. We calculated the sum of runs across all attitude items and respondents and did so also for 10,000 permuted datasets in which attitude scores were randomly reordered within individual respondent record. Panel (*B*) in Fig. 4 shows that the sum of runs in the original data is lower than in all $10^{4}$ permuted datasets. To test whether the effect we found was driven by a subset of the items, we performed the same analysis on a per-item basis. In this analysis, we excluded items for which there were fewer than 1,000 respondents who answered in at least three waves of the survey, which resulted in low power. All 25 remaining items failed the exchangeability test at the P<0.001 level.^¶¶^ With the exchangeability property of the data disconfirmed, the simplicity argument in favor of a heterogeneity (i.e., Settled Dispositions Model) over a reinforcement (i.e., Settled Dispositions Model twin) interpretation is invalidated.

Having refuted exchangeability in two datasets, we next ask how readily one should expect the exchangeability property to hold in empirical datasets and thus to produce a verdict in favor of the heterogeneity model, assuming that the true generating mechanism is actually a pure heterogeneity mechanism. To do this, we test this property in a dataset from a controlled experiment in which experimental conditions remained constant over time and reinforcement was excluded by design. If under these favorable conditions exchangeability fails, then it is unlikely for most empirical datasets to satisfy this property. A simplicity argument will then rarely be possible in practice.

Specifically, we test for exchangeability in data from the control condition of Music Lab (78), an online experiment in which participants were assigned to nine distinct experimental groups–“worlds”–and invited to download one or more of 48 unfamiliar songs. In eight worlds, participants saw counts of prior downloads by earlier participants in their world. In these eight worlds, both a heterogeneity model and a reinforcement model are appropriate: It is mechanically possible that download behavior is mainly driven by song quality and also that it is mainly driven by reinforcement dynamics following idiosyncratic downloads of early participants. Our focus instead is on world 9, wherein individuals could not observe others’ prior downloads. In this control condition, only a song’s intrinsic appeal and screen location, which was randomized, can determine song success. A stationary heterogeneity model should be a good approximation of behavior in the control condition, because all participants are confronted with an identical environment. The only conceivable source of nonstationarity is that participants with different song preferences do not enter the experiment in random order. The results of the exchangeability test (Fig. 4*C*; see *Materials and Methods*) show that the independence condition of Music Lab nonetheless fails the test. Hence, even though the environment was controlled and constructed to produce data for which a stationary heterogeneity model should be a good approximation, the statistical test finds that the data cannot have come from such a model.

We conclude that in practice exchangeability is a property that data may often fail even when stationary heterogeneity is a reasonable approximation of the data generating process. More generally, in contexts in which social science models fit data only approximately and not perfectly, model simplicity cannot be effectively used to differentiate between a heterogeneity model and its exact reinforcement twin.

## Discussion

Our results provide an explanation for a puzzling lack of empirical support for reinforcement in research contexts where reinforcement is strongly suspected on the basis of prior knowledge: Reinforcement processes may generate variability in data that appears to have been produced by structural differences in an unobserved trait. Hence, data used in studies that fit models that base outcomes on preexisting individual qualities equally fit predictions from a reinforcement model in which all individuals ex ante have an equal propensity for high or low values. Hybrid models that combine both elements of heterogeneity and reinforcement are also fully confounded with pure heterogeneity and pure reinforcement models. As we have demonstrated here, this explains how studies on success in academia may fail to find support for cumulative advantage in observational data even when quasi-experimental evidence finds it supported. It similarly explains how panel studies can find personal culture to be mostly stable over the life course even when individuals do flexibly update their values and beliefs as their environments change. The generality of heterogeneity and reinforcement as basic generative forces across a wide range of scientific contexts suggests there may be many other literatures where systematic differences between individuals or groups in longitudinal records produced by reinforcement run the risk of being taken as evidence for unobserved heterogeneity. The risk of misattribution is particularly high in research contexts where strong priors for reinforcement exist.

Our results also have implications for analyses of individual time series in which reinforcement is thought to play an important role in generating temporal success patterns. For example, the hot hand effect is the conjecture that athletes are more likely than baseline to make a successful shot after a series of successful shots. Statistical testing has revolved around evaluating stationarity in individual player records, with recent analyses (79) reversing the classic result of no significant deviation (80). Forms of reinforcement may be missed in such analyses. Stationarity in binary streak data does not disprove reinforcement but is instead consistent with reinforcement on the cumulative average success over the course of a game or shooting experiment, as we exemplified with the Pólya urn model. The deviations from randomness found in analysis of streak data (79) might only evidence a portion of reinforcement effects that operates on the immediate past. Reinforcement that operates on the entire game cannot be inferred by comparing data across games either, because of the confounding with alterations in game conditions.

Previous studies have shown confounding of heterogeneity with reinforcement or duration dependence for models of discrete events (13–16, 20, 21). Our mathematical results show generic confounding, in the sense that for any type of longitudinal data there is always a pure reinforcement model as well as a pure heterogeneity model that could have generated it. This includes data with varying differences between units, e.g., converging or diverging outcome sequences, as well as cases in which the population size is not constant, e.g., new units are added with time.

One principle for selecting between generative models that make the same statistical predictions is simplicity, or parsimony. In particular, it is a common modeling technique to employ simplifying assumptions (e.g., stationarity, linearity, independence relations, etc.) that make the model more parsimonious and at the same time identifiable and justify the use of the assumptions a posteriori based on statistical fit. However, our results suggest that this will generally lead to misguided inferences, unless the specific assumptions made are tested empirically. Indeed, the generic confounding of heterogeneity and reinforcement demonstrated in this paper implies that by imposing arbitrary restrictions or parametric forms, one necessarily ends up attributing interpersonal variability to one of the two mechanisms when this could have been generated by the other. An analogous issue has been previously noted about the use of restrictions on the form of effects in attempts to solve the age-period-cohort problem (81) and to identify heterogeneity and duration dependence in hazard models (82). We have demonstrated for the particular case of stationarity, which may be taken to favor time-constant heterogeneity models, that in practice social science data are unlikely to satisfy this property. Indeed, we found that citation data and personal culture data are highly nonexchangeable, despite the fact that stationary heterogeneity models can provide a good fit to the cross-sectional statistics. We found stationarity rejected even under favorable circumstances, in a controlled experiment where experimental conditions remained stable over time and reinforcement was precluded by design (78).

We propose two ways out of the generic confoundedness of reinforcement and unobserved heterogeneity in longitudinal data. The first strategy is to bound the role of reinforcement from below by isolating a random shock and measuring its reverberating impact on an outcome variable. Regression discontinuity designs and natural experiments can thus be used to examine effects of a randomly induced change in the outcome on later observations of the outcome (41–43, 47, 83–86). The second is observed heterogeneity: In many settings, some of the heterogeneity in the population will be observable, e.g., measured individual demographics. If systematic differences between units correlate with fixed features of these units, then they imply a lower bound on heterogeneity. For example, in all three cases we discussed, empirical studies worked with data on individuals for whom a range of observed features can be introduced into the analysis. In such cases, it is still possible that the variability is not produced by the observed traits but by other unobserved traits correlated with them. It is also possible that heterogeneity and reinforcement interact to produce interpersonal differences, such as in the limited differences model, where small gender differences are reinforced (48). Effects of fixed traits, nonetheless, establish heterogeneity as a (co-)determinant of the explained variance.

## Materials and Methods

### Figs. 1 and 2.

Simulations were run in MATLAB (2023a) and figures generated in R (4.2.2). For the Q-model and its twin, the log-citation numbers $Y_{n}$ were simulated, and then the cumulative number of citations was calculated as $\sum_{k=1}^{n}e^{Y_{k}}$. For the model of sexual contacts and its twin, we simulated interevent times $\tau_{k+1}=t_{k+1}-t_{k}$ and then we calculated the number of events occurring in the interval [0,t] for t=1,…,10. In the case of the original model of sexual contacts, we first drew a heterogeneity parameter κ, which remained constant for all $\tau_{j}$’s of a single trajectory, and simulated $\tau_{k+1}$ as an exponential random variable with rate $\kappa \cdot \pi_{k}$, where $\pi_{k}$ is as described in Fig. 2*C*. For its twin, we simulated i.i.d. exponential random variables with unit rate $z_{1},z_{2},\ldots$ and set $\tau_{k+1}=s_{k+1}\left(z_{k+1}\right)$, where

[8] $$s_{k+1}\left(t\right)=\left[\alpha +\overset{k-1}{\sum_{j=0}}\;\pi_{j}\cdot \left(t_{j+1}-t_{j}\right)\right]\cdot \frac{e^{\frac{t}{k+\alpha }}-1}{\pi_{k}}.$$

(See *SI Appendix*, *Extended Methods* for a justification.)

### Fig. 3.

We used the disambiguated data from ref. 30 (*SI Appendix*, *Extended Methods*). We fit a log-normal distribution with μ=2.79 and $\sigma^{2}=1.39$. The parameter values shown in the legend for each of the models are the ones that give rise to this distribution for $c_{10}$. For the Q-model, we have $\mu_{T}=\mu$ and $\sigma_{T}^{2}+\sigma_{X}^{2}=\sigma^{2}$. For the twin model, we used *Proposition* 2 to obtain the parameters a,b,c from $\mu_{T}$, $\sigma_{T}^{2}$, and $\sigma_{X}^{2}$.

### Fig. 4.

For the Music Lab data (Panel *C*), for each of the two experiments reported in ref. 78 and each song, we extracted a binary sequence whose n-th entry indicated whether the n-th user of world 9 downloaded the song. We calculated the number of runs and summed across songs and across the two experiments. We also did so for 10,000 datasets with the order of the users shuffled. The methods for Panels (*A* and *B*) are described in the main text. *P*-values for a two-sided test in the personal culture data were estimated as 2·min{x,1−x}, where x is the proportion of permuted datasets that had fewer runs than the original dataset.

All code and data are available online at: https://github.com/Lsage/Cumulative-advantage/.

## Supplementary Material

Appendix 01 (PDF)

## Data Availability

Citation counts of research papers have been deposited in GitHub (https://github.com/Lsage/Cumulative-advantage/). Previously published data were used for this work (77, 78).

## Footnotes

^*^To be more precise, the ball colors are assumed *conditionally* independent, given the value of p. If one does not condition on p, then the colors of different balls are not statistically independent, because they are linked through the value of p. This alternative model is equivalent to an empty random effects probit model with zero intercept, in which the random effects term has the same variance as the error term.

^†^Here, we assume that infinitely many papers are produced by each individual, i.e., we ignore the question of productivity.

^‡^We use the shape-scale parameterization of the Gamma distribution, that is, $Gamma\left(l,\theta \right)∼\frac{1}{\Gamma \left(l\right)\theta^{l}}x^{l-1}e^{-x/\theta }$.

^§^We use the notation $\left\{Y_{t}\right\}$ to refer to the entire process, and $Y_{t}$ to refer to the value at time t.

^¶^In ref. 29, the $\pi_{j}$’s have a certain parametric form, but our result is independent of these choices.

^#^This is the hazard function introduced in ref. 63, p. 231. By equation 7.2.3 and proposition 7.2.IV in the same reference, this uniquely determines the process.

^‖^More generally, the $Y_{n}$’s may take values in an arbitrary Borel space (75). This will be our default assumption for all random variables throughout the text.

^**^Measurability is a technical term, which is the minimal requirement to guarantee that $Y_{n}$ is a random variable.

^††^In fact, the proof of theorem 4 shows that even a deterministic heterogeneity model (i.e., one without the luck factor $X_{n}$) can reproduce the probability distribution of $\left\{Y_{n}\right\}$. All randomness, correlations between observations, etc., can be attributed to an unobserved variable U that is inherent to the individual unit we are observing.

^‡‡^A permutation is finite if it leaves all indices fixed except a finite number of them.

^§§^This significance level results from the number of permutations we ran ($10^{4}$) for a two-sided test. Fig. 4*A* visually demonstrates that the actual P-value is likely orders of magnitude smaller.

^¶¶^Among the 27 items that did not satisfy our power criterion, only 6 were found to deviate significantly from exchangeability at the 0.05 level; see *SI Appendix*, Table S1 and Fig. S1.
