# Supplementary material for: Reinforcement generates systematic differences without heterogeneity
Source: Proc Natl Acad Sci U S A. 2025 Jun 6;122(23):e2408163122. doi: 10.1073/pnas.2408163122 (PMC12167982; doi:10.1073/pnas.2408163122)
Supplement: Supplementary file 1 — Appendix 01 (PDF) [file pnas.2408163122.sapp.pdf]

Supporting Information for

Reinforcement generates systematic differences without heterogeneity

Alexandros Gelastopoulos, Lucas Sage, and Arnout van de Rijt

Corresponding Author: Arnout van de Rijt.
E-mail: arnout.vanderijt@eui.eu

This PDF file includes:

Supporting text
Figs. S1 to S3
Table S1
SI References

Supporting Information Text

Extended methods

Citation data. We used the disambiguated data from (1). The data only contained a list of publications identified by their doi and the associated authors identified by an id. The articles correspond to all the papers published in the journals of the American Physical Association up until 2009. For more information about the data and the disambiguation procedure we refer the reader to the original article. We collected the number of citations each article received in the 10 years following the publication date using OpenAlex (2). Following (1), we restricted the sample to authors with a career span of at least 20 years (defined as the time span between the date of their first and last publication in data), and we defined c_n as the number of citations of the n-th publication of a researcher in the 10-year period following the publication. In order to discount for variation that is due to different researchers having different number of publications (i.e., the population varies with n), we restricted our analysis to the first 50 publications of researchers that had at least 50 publications.

Attitudes data. We used data from the Panel Study of Income Dynamics 1968-2019. We followed (3) and selected variables from the core study plus the Child Development Supplement (CDS) and Transition to Adulthood Supplement (TAS). We used the replication material of (3)'s to process the data (4). We used items that appeared in at least three waves of the survey and, for each item, we excluded individuals who responded in fewer than three waves. Table S1 summarizes the dataset and fig. S1 plots the p-values against the effective sample size. The effective sample size is defined as the number of individuals who responded in at least three waves of the study and whose answers were neither all identical nor all different, so that permuting their order could alter the number of runs.

Simulation of inter-event times for the reinforcement twin of the sexual contacts model. In the reinforcement twin of the sexual contacts model, the instantaneous rate of Y'_t depends both on the history of the process (number m of events so far and time of events $t_1, \ldots, t_m$), and explicitly on time, specifically given by (Eq. 4 in the main text)

$$\lambda_{m+1}(t \mid t_1, \ldots, t_m) = \frac{\pi_m \cdot (m+\alpha)}{\pi_m \cdot t - \sum_{j=1}^{m} (\pi_j - \pi_{j-1}) t_j + \alpha}, \qquad [1]$$

We simulate the $(m+1)$-th inter-event time $\tau_{m+1} = t_{m+1} - t_m$ as follows: we first note that in the interval $[t_m, t_{m+1}]$, Y'_t behaves like a non-homogeneous Poisson process with rate given by eq. (1) with m and $t_1, \ldots, t_m$ remaining constant. Therefore, we may consider a time translation that takes t_m to 0, and simulate τ_{m+1} as the *first* event time of a non-homogeneous Poisson process Y''_t with rate given by eq. (1) with $t + t_m$ in place of t, that is with rate

$$g_{m+1}(t) = \frac{\pi_m \cdot (m+\alpha)}{\pi_m \cdot (t + t_m) - \sum_{j=1}^{m} (\pi_j - \pi_{j-1}) t_j + \alpha} = \frac{\pi_m \cdot (m+\alpha)}{\pi_m \cdot t + \sum_{j=0}^{m-1} \pi_j (t_{j+1} - t_j) + \alpha}. \qquad [2]$$

(To check this equation, note that at $t = 0$ it takes the same value as $\lambda_{m+1}(\cdot \mid t_1, \ldots, t_m)$ does at $t = t_m$.)

To simulate the first event time of Y''_t, we use the following lemma:

Lemma 1. *Let Y_t be a non-homogeneous Poisson process with bounded, strictly positive rate $\lambda(t)$. Define $a(t) = \int_0^t \lambda(u)du$ and its inverse $s(t) = a^{-1}(t)$. The first event time of Y_t has the same distribution as $s(z)$ where z is a unit-rate exponential random variable.*

Proof. The function $a(t)$ is continuous, strictly increasing (so that its inverse is well-defined), and it gives the expected number of events in the interval $[0, t]$. By Corollary 4.7.8 in (5), if T_1 is the first event time for Y_t, then $z = a(T_1)$ is the first event time for a homogeneous Poisson process with unit rate. The latter equation can be rewritten as $T_1 = s(z)$. Moreover, z is exponential with unit rate by a basic property of the homogeneous Poisson process. □

In our case, we have

$$a_{m+1}(t) = \int_0^t g_{m+1}(u)du = (m+\alpha) \cdot \ln\left(1 + \frac{\pi_m t}{\sum_{j=0}^{m-1} \pi_j (t_{j+1} - t_j) + \alpha}\right), \qquad [3]$$

and for its inverse

$$s_{m+1}(t) = \left[\sum_{j=0}^{m-1} \pi_j (t_{j+1} - t_j) + \alpha\right] \cdot \frac{e^{\frac{t}{m+\alpha}} - 1}{\pi_m}. \qquad [4]$$

Thus, τ_{m+1} may be simulated as $s_{m+1}(z_{m+1})$, where z_{m+1} is a unit-rate exponential random variable.

Variable	N ind. original	N ind. with ≥ 3 obs.	Effective sample size	Obs.	Mean	Sd	Min	Max	p-value
cds_bothparentscare	4516	810	467	2599	3.4424779	0.5960966	1	4	0.0868
cds_daddevelopment	4515	808	445	2593	3.4168916	0.5978163	1	4	0.0018
cds_dadoldkidbetter	2426	638	311	1914	2.2032393	0.7739688	1	4	0.0536
cds_dadtooinvolved	2424	620	325	1860	1.8752688	0.6531516	1	4	0.0598
cds_daycareok	4503	789	428	2534	2.3642463	0.7909877	1	4	0.0540
cds_fatherhoodfulfill	2400	583	294	1749	3.2784448	0.6179047	1	4	0.6278
cds_fatherinteractchild	4517	805	415	2583	3.5512969	0.5576568	1	4	0.4972
cds_firstyears	2438	638	314	1914	3.4409613	0.5987566	1	4	0.5028
cds_girlboyequal	4519	807	396	2593	3.4874663	0.6442536	1	4	0.6950
cds_husbandcareerimport	4513	802	463	2573	1.8087835	0.7131081	1	4	0.0698
cds_mostimportantchild	4522	808	380	2591	3.0690853	1.1140787	1	5	0.9116
cds_motherfatherequal	2437	643	318	1929	3.0995334	0.6697717	1	4	0.0106
cds_motherhoodfulfill	2428	647	338	1941	3.4822257	0.5949256	1	4	0.6086
cds_mothernotft	4504	803	441	2576	2.3027950	0.8191447	1	4	0.0030
cds_preschoolsuffer	4505	795	416	2551	2.0889847	0.7753052	1	4	0.0204
cds_sharehousehold	4518	812	459	2607	3.4802455	0.6616141	1	4	0.1022
cds_traditionalbest	4502	793	421	2548	2.2672684	0.8394920	1	4	0.1514
cds_workingmotherssame	4515	799	463	2567	3.0985586	0.7955607	1	4	0.5218
gettingbetter	12416	9039	5895	40913	2.7113876	1.8689206	1	5	0.0000
lifesatis	25380	17547	12678	89534	2.1519088	0.8385788	1	5	0.0000
likejobormoney	12464	9152	3913	42340	1.7154228	1.5038793	1	5	0.0000
opinionimport	12503	9209	5944	42670	2.1958988	1.5002049	1	5	0.0000
satisfiedself	12507	9228	3928	43007	1.7249052	1.4757362	1	5	0.0000
savingspending	12487	9196	6343	42654	2.9510714	1.7872500	1	5	0.0000
tas_belongcommunity	3943	2099	1330	8462	3.7284330	1.9293740	1	6	0.0000
tas_contributesociety	3940	2100	1388	8462	4.4334673	1.4621264	1	6	0.0000
tas_divorcehurts	2875	1751	945	6772	5.8007974	1.5723705	1	7	0.0000
tas_divorcesuccess	2875	1751	974	6774	6.1597284	1.2613093	1	7	0.0000
tas_fathertimeoff	2874	1751	999	6772	5.3492321	1.6374668	1	7	0.0000
tas_importrel	3934	2098	1239	8450	2.7904142	1.4763622	0	5	0.0000
tas_importspirit	3935	2102	1259	8471	2.1456735	1.8340940	0	5	0.0000
tas_lifesatisfact	3789	1197	702	4182	2.2364897	0.7953933	1	5	0.0002
tas_mothersnodemanding	2875	1748	1042	6762	3.9375924	1.8193712	1	7	0.0000
tas_peoplearegood	3941	2099	1355	8458	3.6853866	1.5324257	1	6	0.0000
tas_religpref0509	1609	554	172	1662	0.6973526	0.7808547	0	3	0.2834
tas_religpref1115	2717	808	190	2424	0.8783003	0.6692030	0	3	0.0000
tas_singleparentsok	2875	1750	953	6771	6.2075026	1.2238073	1	7	0.0000
tas_singleparentsuffer	2875	1751	1068	6774	3.6842338	1.9917731	1	7	0.0000
tas_singlewomenkids	2875	1751	753	6771	1.6967952	1.3584095	1	7	0.0000
tas_societybetter	3939	2100	1355	8456	2.8681410	1.5864587	1	6	0.0000
tas_societymakessense	3939	2098	1312	8454	3.0114739	1.6169971	1	6	0.0000
tas_spritual	3934	2090	922	8410	2.6085612	1.9614365	1	5	0.0000
thinkfuture	12510	9241	5746	43090	3.4016477	1.9091414	1	5	0.0000
trustothers	12511	9238	5621	43009	2.5542561	1.6781989	1	5	0.0000
undeserved	12225	8481	5212	36950	3.1766982	1.8941468	1	5	0.0000
tas_religpref0509_2	1609	554	142	1662	0.3387485	0.4734267	0	1	0.6468
tas_religpref0509_3	1609	554	40	1662	0.1594465	0.3662021	0	1	0.5434
tas_religpref1115_2	2717	808	176	2424	0.5684818	0.4953902	0	1	0.0000
tas_religpref1115_3	2717	808	71	2424	0.1394389	0.3464755	0	1	0.5974
cds_mostimportantchild_1	4522	808	236	2591	0.1570822	0.3639485	0	1	0.1116
cds_mostimportantchild_3	4522	808	421	2591	0.5488228	0.4977067	0	1	0.0608
cds_mostimportantchild_4	4522	808	301	2591	0.1771517	0.3818707	0	1	0.1172

Table S1. Descriptive statistics and p-value for 52 attitude variables in the PSID data. Columns: 1. variable name, 2. number of respondents, 3. number of respondents who responded in at least three waves of the survey, 4. number of respondents from column 3 excluding those whose answers were either all identical or all different (so shuffling the order has no effect on the number of runs) 5. number of observations for respondents of column 3, 6-9. Mean, Standard deviation, scale minimum and scale maximum, 10. estimated p-value for two-sided runs test, based on 10,000 permuted datasets.

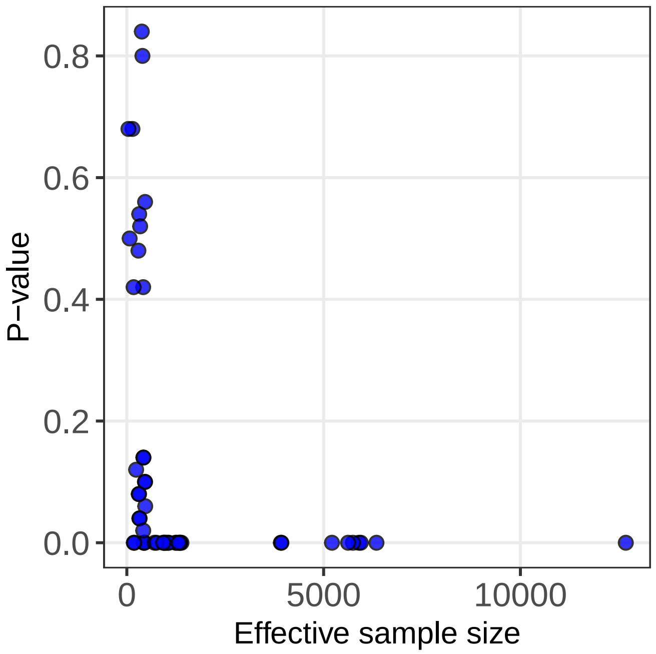

Fig. S1. p-values vs effective sample size for the 52 items in table S1. All items with effective sample size > 500 have highly significant p-value.

Proofs of Propositions 2 and 3

We denote by $\mathcal{U}(0, 1)$ a uniform probability distribution on the interval $[0, 1]$.

Proof of Proposition 2. The variables $Y_1, \ldots, Y_n$ can be thought of as noisy observations of the variable T, which has prior distribution $\mathcal{N}(\mu_T, \sigma_T^2)$, and the noise is distributed according to $\mathcal{N}(0, \sigma_X^2)$. By a standard result in Bayesian estimation (see for example (6, Ch. 4, Example 2.2)), conditioned on $Y_1, \ldots, Y_n$, the posterior distribution of T is normal with mean

$$\frac{\frac{\sigma_X^2}{\sigma_T^2} \cdot \mu_T + \sum_{k=1}^{n} Y_k}{n + \frac{\sigma_X^2}{\sigma_T^2}}$$

and variance $\frac{\sigma_X^2}{n + \frac{\sigma_X^2}{\sigma_T^2}}$. Therefore, conditioned on $Y_1, \ldots, Y_n$, the variable $Y_{n+1} = T + X_{n+1}$ is distributed normally with the same mean and with variance

$$\frac{\sigma_X^2}{n + \frac{\sigma_X^2}{\sigma_T^2}} + \sigma_X^2 = \sigma_X^2 \cdot \left(1 + \frac{1}{n + \frac{\sigma_X^2}{\sigma_T^2}}\right).$$

This is the same as the conditional distribution of Y'_{n+1}, conditioned on $Y'_1, \ldots, Y'_n$, if we set $a = \mu_T$, $b = \sigma_X^2$, and $c = \frac{\sigma_X^2}{\sigma_T^2}$ (by Eqs. 1 and 2 in the main text).

Since the conditional distribution of Y_{n+1} given $Y_1, \ldots, Y_n$ is the same as the conditional distribution of Y'_{n+1} given $Y'_1, \ldots, Y'_n$ for all $n \in \mathbb{N}$, the joint distributions of $Y_1, Y_2, \ldots$ and $Y'_1, Y'_2, \ldots$ are identical. $\square$

Proof of Proposition 3. By Proposition 7.2.IV and Eq. 7.2.3 in (7), a regular point process on $[0, \infty)$ is uniquely determined by its conditional intensity function, which is in turn determined by the (conditional) hazard rates $\lambda_m(t \mid t_1, \ldots, t_{m-1})$. Therefore, it is enough to show that the hazard rates for the mixed model of (8) are given by eq. (1).

First note that, by definition, conditioned on the heterogeneity parameter $\kappa = x$, on $Y_t = m$, and on the times of the first m events $t_1, \ldots, t_m$, the probability that there is a new event for the process $\{Y_t\}$ in the interval $(t, t + \Delta t]$ is $x\pi_m \Delta t + o(\Delta t)$, where $o(\Delta t)$ denotes terms that go to 0 faster than Δt. Therefore, if we only condition on $Y_t = m$ and the event times $t_1, \ldots, t_m$, the probability of the same event is given by

$$\begin{aligned} &\int_0^\infty x\pi_m \, f_\kappa(x \mid m, t_1, \ldots, t_m) dx \cdot \Delta t + o(\Delta t) \\ =&\pi_m \cdot \int_0^\infty x \, f_\kappa(x \mid m, t_1, \ldots, t_m) dx \cdot \Delta t + o(\Delta t), \end{aligned} \quad [5]$$

where $f_\kappa(x \mid m, t_1, \ldots, t_m)$ is the conditional probability density of κ given m and $t_1, \ldots, t_m$. It follows that the $(m+1)$-st hazard rate is

$$\lambda_{m+1}(t \mid t_1, \ldots, t_m) = \pi_m \cdot \int_0^\infty x f_\kappa(x \mid m, t_1, \ldots, t_m) dx, \qquad [6]$$

The conditional density $f_\kappa(x \mid m, t_1, \ldots, t_m)$ is given by Bayes' rule:

$$f_\kappa(x \mid m, t_1, \ldots, t_m) = \frac{g(x) \cdot L_t(m, t_1, \ldots, t_m; x)}{\int_0^\infty g(y) \cdot L_t(m, t_1, \ldots, t_m; y) dy}, \qquad [7]$$

where g is the prior distribution of κ, and $L_t(m, t_1, \ldots, t_m; x)$ is the likelihood of $Y_t = m$ and the first m events occurring at times $t_1, \ldots, t_m$, assuming $\kappa = x$. Thus, eq. (6) becomes

$$\lambda_{m+1}(t \mid t_1, \ldots, t_m) = \pi_m \cdot \frac{\int_0^\infty y \cdot g(y) \cdot L_t(m, t_1, \ldots, t_m; y) dy}{\int_0^\infty g(y) \cdot L_t(m, t_1, \ldots, t_m; y) dy}, \qquad [8]$$

Recall that g is $Gamma(\alpha, 1/\alpha)$-distributed, i.e.,

$$g(x) = \frac{\alpha^\alpha}{\Gamma(\alpha)} x^{\alpha-1} \cdot e^{-\alpha x}, \qquad [9]$$

while the likelihood $L_t(m, t_1, \ldots, t_m; y)$ is given by (see Proposition 7.2.III in (7), noting that given $\kappa = x$, the hazard rates are simply $\lambda_j = x \cdot \pi_j$ by definition)

$$L_t\left(m, t_1, \ldots, t_m; x\right) = x^m \prod_{j=0}^{m-1} \pi_j \cdot e^{-x \cdot \sum_{j=0}^m \pi_j (t_{j+1} - t_j)}, \qquad [10]$$

where $t_{m+1} = t$ and $t_0 = 0$. Substituting the last two equations into eq. (8) and simplifying, and denoting $C = \sum_{j=0}^m \pi_j (t_{j+1} - t_j)$, we get

$$\lambda_{m+1}(t \mid t_1, \ldots, t_m) = \pi_m \cdot \frac{\int_0^\infty y^{m+\alpha} \cdot e^{-y(C+\alpha)} dy}{\int_0^\infty y^{m+\alpha-1} \cdot e^{-y(C+\alpha)} dy}. \qquad [11]$$

We apply the product rule to the integral in the numerator to get

$$\begin{aligned} \int_0^\infty y^{m+\alpha} \cdot e^{-y(C+\alpha)} dy &= \frac{1}{C+\alpha} \cdot \int_0^\infty y^{m+\alpha} \left[e^{-y(C+\alpha)}\right]' dy \\ &= \frac{m+\alpha}{C+\alpha} \cdot \int_0^\infty y^{m+\alpha-1} e^{-y(C+\alpha)} dy. \end{aligned} \qquad [12]$$

Substituting this into eq. (11), we get

$$\begin{aligned} \lambda_{m+1}(t \mid t_1, \ldots, t_m) &= \pi_m \cdot \frac{m+\alpha}{C+\alpha} \\ &= \frac{\pi_m \cdot (m+\alpha)}{\sum_{j=0}^m \pi_j (t_{j+1} - t_j) + \alpha}. \end{aligned} \qquad [13]$$

Recalling that $t_{m+1} = t$ and $t_0 = 0$ and regrouping the terms in the denominator, we get

$$\lambda_{m+1}(t \mid t_1, \ldots, t_m) = \frac{\pi_m \cdot (m+\alpha)}{\pi_m t - \sum_{j=1}^m (\pi_j - \pi_{j-1}) t_j + \alpha}, \qquad [14]$$

which is the same as eq. (1), as required. □

Examples of reinforcement models and heterogeneity models

Here we show how some standard models of reinforcement can be described within our framework, i.e., written in terms of the definition we have given in the main text. We also give an example of a model from the literature (the random-typing monkey) that can be expressed as a heterogeneity model. In what follows, $\mathcal{U}(0, 1)$ denotes the uniform probability distribution on the interval $[0, 1]$.

Pólya urn. In the standard Pólya urn, the probability that a red ball is added at step n is equal to $\frac{n_R}{n-1+K}$, where n_R is the number of red balls currently in the urn (at time $n-1$) and K is the initial number of balls in the urn. Letting $Y_n = 1$ denote the fact that a red ball is drawn at step n (and $Y_n = 0$ otherwise), then n_R is equal to $\sum_{k=1}^{n-1} Y_k + K_R$, where K_R is the initial number of red balls. Therefore, the probability of the event $Y_n = 1$ is equal to $\frac{\sum_{k=1}^{n-1} Y_k + K_R}{n-1+K}$.

We now define a reinforcement model, according to the definition given in the main manuscript, which is equivalent to the Pólya urn just described. Let $\{X_n\}$ be an i.i.d. sequence of $\mathcal{U}(0,1)$ random variables and define $Y_n = g_n(Y_1, \ldots, Y_{n-1}, X_n)$, where

$$g_n(Y_1, \ldots, Y_{n-1}, X_n) = \begin{cases} 1, & \text{if } X_n < \frac{\sum_{k=1}^{n-1} Y_k + K_R}{n-1+K} \\ 0, & \text{otherwise.} \end{cases} \qquad [15]$$

Clearly, conditioned on $Y_1, \ldots, Y_{n-1}$, the probability of the event $Y_n = 1$ is equal to $\frac{\sum_{k=1}^{n-1} Y_k + K_R}{n-1+K}$, exactly as required. We thus see that the Pólya urn model fits our definition of a reinforcement model.

Preferential attachment. A preferential attachment model is similar to a Pólya urn, except that balls of new colors may enter the urn. For example, Simon (9) describes the generation of a text by adding words one by one, with the probability of adding a given word being proportional to the occurrences of that word in the text, except that there is a probability $\alpha \in (0,1)$ that an entirely new word appears. If we focus on a particular word that is already present in the text,* the probability of a new occurrence of this word at step n is then $(1-\alpha) \cdot \frac{n_R}{n-1+K}$, where n_R is the number of occurrences of that word so far, and K is the number of words in the text at time 0. Similarly, Hoppe (10) considers an alternative urn scheme which allows for new color entrants, but unlike Simon's model, the probability of a ball being of an entirely new color decreases with time. Specifically, the probability of repeating an existing color i is $\frac{n_i}{n+\alpha}$, while the probability of introducing a new color is $\frac{\alpha}{n+\alpha}$.† Finally, in the Blackwell-MacQueen urn, the colors have identities at the outset (even before they are introduced), and a ball of color i is introduced with probability $\frac{\alpha p_i + n_i}{n+\alpha}$, where the p_i's are non-negative constants with $\sum_i p_i = 1$. These models can be expressed in terms of our definition of a reinforcement model in a way similar to a Pólya urn.

Barabási and Albert (11) describe the growth of a network in which at each step a new node is added together with a number of links, which connect it to existing nodes. The connecting nodes are chosen randomly, with probability proportional to their current degree, i.e., the number of existing links to these nodes.

For simplicity, we assume here that for each node that enters, only one link is created. Assuming that the network at time $n = 0$ contains m_0 links, then at time n it has $n + m_0$ links, and because each link contributes to the degree of two nodes, the sum of the degrees is $2(n + m_0)$. A node that enters at time n' will therefore have zero probability of obtaining links for $n < n'$, probability equal to 1 at time $n = n'$, and probability $\frac{d_n}{2(n+m_0)}$ of obtaining a link at any $n > n'$, where D_n is its degree. Again, this model can be cast in terms of our definition of a reinforcement model in a way similar to a Pólya urn. We note, however, that the resulting probability distribution of d_n is not the distribution of the degree of a randomly chosen node at time n, because nodes enter at different times. What the distribution of d_n tells us is the probability that the degree of the node entering at the pre-specified time n' will take any given value at time n.

To describe the growth of the network *as a whole*, we may define the network G_n at time n as an $(m_0 + n)$-tuple of positive integers, $G_n = (d_n^1, \ldots, d_n^{m_0+n}) \in \mathbb{N}^{m_0+n}$, where d_n^i denotes the degree of node i at time n.‡ At time $n+1$, the (m_0+n+1)-th node enters and its degree will be equal to 1 with certainty. The degree of one other node will increase by 1, and the probability that this node will be node i is d_n^i / D_n, where $D_n = \sum_{j=1}^{m+n_0} d_j = 2(n + m_0)$ is the sum of degrees.

To express this in the form of Eq. 1 of the main text, let G'_n denote the vector obtained by appending a 1 to G_n, that is, $G'_n = (d_n^1, \ldots, d_n^{m_0+n}, 1) \in \mathbb{N}^{m_0+n+1}$. Also, let X_n be $\mathcal{U}(0,1)$-distributed and define K_n to be equal to $k \in \{1, \ldots, m_0 + n\}$ if $X_n \in \left[\sum_{j=1}^{k-1} d_n^j / D_n, \sum_{j=1}^{k} d_n^j / D_n\right)$. It follows that K_n takes the value k with probability d_n^k / D_n. Let $E_n^i \in \mathbb{N}^{m_0+n+1}$ denote the vector consisting of all 0's, except that at position i it has a 1. We define,

$$G_{n+1} = g_{n+1}(G_n, X_n) := G'_n + E_n^{K_n}. \qquad [16]$$

It follows that G_{n+1} differs from G_n only in two ways: it has one additional node with degree 1, and the degree of another node increased by 1. Moreover, the latter node was node $K_n = k$ with probability d_n^k / D_n, as required.

* In this model, the way that newly appearing words are chosen is not specified. One can say that words do not exist before their first appearance. Thus, specifying the probabilities of the first appearance of a specific word in this model is impossible without further assumptions.

† In Hoppe's urn $K = 1$.

‡ Because the $(m_0 + n)$-th node has just entered, its degree is equal to 1, that is, $d_n^{m_0+n} = 1$.

Price's urn model. Price (12) defines the following urn model: An urn with 1 black and 1 red ball is sampled repeatedly, with red balls interpreted as successes. Whenever a red ball is drawn, it is returned together with another red ball. The first time a black ball is drawn, we stop drawing balls altogether. Interpreting $Y_n = 1$ as a success (red ball drawn) and $Y_n = 0$ as a failure (black ball drawn or no draw), we may define an equivalent generative model as follows: X_n are i.i.d. $\mathcal{U}(0,1)$ random variables and $Y_n = g_n(Y_1, \ldots, Y_{n-1}, X_n)$, where

$$g_n(Y_1, \ldots, Y_{n-1}, X_n) = \begin{cases} 1, & \text{if } Y_1 = \ldots = Y_{n-1} = 1 \\ & \text{and } X_n < \frac{n}{n+1}, \\ 0, & \text{otherwise.} \end{cases} \quad [17]$$

This is again an instance of a reinforcement model according to our definition.

Gibrat's Law of proportional effect. Gibrat (13) suggested that the size of firms in terms of number of employees, as well as the size of cities in terms of number of inhabitants, grows on average proportionally to their current size. Specifically, if Y_{n-1} is the size of a firm in year $n-1$, then the size in year n is given by $Y_n = Y_{n-1} \cdot (1 + X_n)$ where $\{X_n\}$ is an i.i.d. sequence of random variables. Thus, this is a special case of a reinforcement model according to our definition, where $g_n(Y_1, \ldots, Y_{n-1}, X_n) = Y_{n-1} \cdot (1 + X_n)$.

The above can be generalized to the case that the X_n's are not i.i.d., even though our definition of a reinforcement model requires an i.i.d. sequence X'_n. To do so, we may choose X'_n to be i.i.d. uniformly distributed in $[0,1]$ and define $g_n(Y_1, \ldots, Y_n, X'_n) = Y_n \cdot (1 + F_n^{-1}(X'_n))$, where F_n^{-1} is the generalized inverse of the cumulative distribution function of X_n. By a standard result in statistics (see inverse transform sampling), the random variable $F_n^{-1}(X'_n)$ has the same distribution as X_n.

Random-typing monkey. Miller (14) showed that a power law distribution for the number of occurrences of different words in a text can be obtained if we simply assume that a monkey presses keys at random. Specifically, we assume that each key is pressed with the same probability, except perhaps from the space key, which is pressed with some fixed probability $\alpha \in (0,1)$. If there are N different keys other than the space key, then the probability of forming a specific word of length k is $\left(\frac{1-\alpha}{N}\right)^k \cdot \alpha$. Because this probability does not depend on previous occurrences of the word, this is a heterogeneity model, specifically a stationary one.

To cast this in the form of a heterogeneity model according to the definition we have given in the main text, we let $\{X_n\}$ be i.i.d. random variables distributed as $\mathcal{U}(0,1)$ and define U to be the length of the word. The function f is given by

$$f(U, X_n) = \begin{cases} 1, & \text{if } X_n < \left(\frac{1-\alpha}{N}\right)^U \cdot \alpha \\ 0, & \text{otherwise.} \end{cases} \quad [18]$$

We get that conditioned on word length $U = k$, the probability of appearance of the word ($f(U, X_n) = 1$) at step n is $\left(\frac{1-\alpha}{N}\right)^k \cdot \alpha$, as required.

The above assumes that U is given, which means that we follow a predefined word. To be consistent with our definition, U must be drawn from a given probability distribution, which here would be equivalent to choosing stochastically a word to track (observe its occurrences). For example, if the word we tracked was chosen uniformly randomly from among all possible words of length at most K, then U would equal $k \in \{1, \ldots, K\}$ with probability $\frac{N^k}{\sum\limits_{l=1}^{K} N^l}$, because there are exactly N^k words of length k.

Conditional variance in the Q-model

Here we check the prediction of the Q-model (and its reinforcement twin) that the conditional variance of the number of citations of the n-th publication of a researcher, given the number of citations to previous publications, decreases with n, specifically being proportional to $1 + 1/(n + c)$ (Eq. 2 in the main text). In the Q-model, this is a consequence of the fact that as researchers make more publications, we have a better estimate of their talent, hence we can better predict the number of citations of their next publication. In contrast, in its reinforcement twin, this property is not justified on theoretical grounds. If this prediction is verified, then it would be an argument for preferring the Q-model over its twin.

The number of citations c_n of the n-th publication of a researcher in the 10-year period following the publication was found as described above (see Extended Methods). Because we are interested in the log-number of citations, and some publications had 0 citations, we added the constant 1 to all publications and estimated $Y_n = \log(c_n + 1)$.

We computed the conditional variance of Y_{n+1} conditioned on $Y_1, \ldots, Y_n$, which by Proposition 2 and Eq. 1 in the main text is equal to the (unconditional) variance of

$$Z_{n+1} := Y_{n+1} - \frac{\sum_{k=1}^{n} Y_k}{n + c}, \quad [19]$$

where different realizations of these sequences correspond to different authors. The constant c in eq. (19) was estimated as $c = \frac{\sigma_X^2}{\sigma_T^2} \approx \frac{1.038}{0.2509} \approx 4.137$, according to Proposition 2 in the main text, where σ_T^2 is the variance in authors' talent T and σ_X^2 is

the variance of $Y_n - T$ (assumed not to depend on n; see the section in the main text that introduces the model). The talent of an author T was estimated as the average of Y_n over their career. The variance of $Y_n - T$ was estimated over all values of n and all authors simultaneously.

Figure S2 shows the variance of Z_n as a function of n. The prediction that $Var(Z_n)$ is monotonically decreasing is not verified. We repeated the calculations for different values of c, to exclude the possibility that the discrepancy was due to estimation error in the value of this parameter. Although for large values of c we find that $Var(Z_n)$ is non-increasing, the shape does not agree with the predicted form $b \cdot \left(1 + \frac{1}{n+c}\right)$, which is a convex function whose (negative) slope is steepest near $n = 0$ (fig. S3). Recall that c is the ratio of the variances of noise over talent. A large value of c would mean that almost all variation in the number of citations is due to noise, with talent playing an insignificant role.

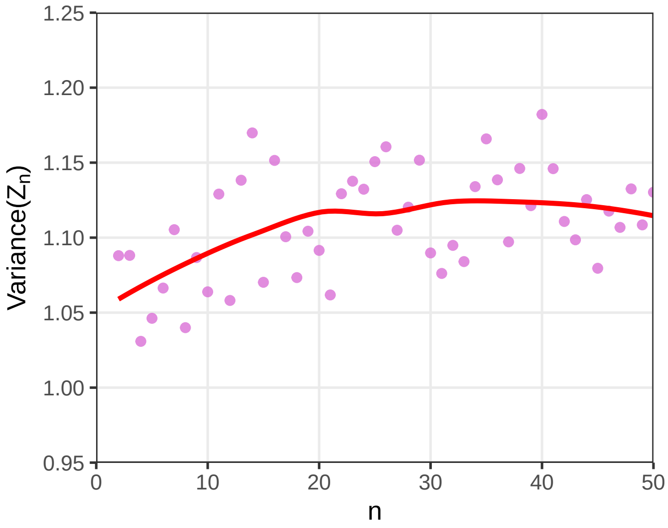

Fig. S2. $Var(Z_n)$ as a function of n (see eq. (19)). Line (red) represents local polynomial regression fitting. The Q-model (as well as its pure reinforcement twin) predicts that $Var(Z_n)$ is monotonically decreasing in n, which is not empirically confirmed.

The code and data are available online at https://github.com/Lsage/Cumulative-advantage/

References

1. R Sinatra, D Wang, P Deville, C Song, AL Barabási, Quantifying the evolution of individual scientific impact. *Science* **354**, aaf5239 (2016).
2. J Priem, H Piwowar, R Orr, Openalex: A fully-open index of scholarly works, authors, venues, institutions, and concepts. *arXiv preprint arXiv:2205.01833* (2022).
3. PM Lersch, Change in personal culture over the life course. *Am. Sociol. Rev.* **88**, 220–251 (2023).
4. PM Lersch, Replication files for "change in personal culture over the life course" (2023).
5. E Cinlar, *Introduction to stochastic processes.* (Courier Corporation), (2013).
6. EL Lehmann, G Casella, *Theory of point estimation.* (Springer Science & Business Media), (2006).
7. DJ Daley, D Vere-Jones, , et al., *An introduction to the theory of point processes: volume I: elementary theory and methods.* (Springer), (2003).
8. BF De Blasio, Å Svensson, F Liljeros, Preferential attachment in sexual networks. *Proc. Natl. Acad. Sci.* **104**, 10762–10767 (2007).
9. HA Simon, On a class of skew distribution functions. *Biometrika* **42**, 425–440 (1955).
10. FM Hoppe, Pólya-like urns and the ewens' sampling formula. *J. Math. Biol.* **20**, 91–94 (1984).
11. AL Barabási, R Albert, Emergence of scaling in random networks. *science* **286**, 509–512 (1999).
12. DdS Price, A general theory of bibliometric and other cumulative advantage processes. *J. Am. society for Inf. science* **27**, 292–306 (1976).
13. R Gibrat, Les inégalits économiques. *Sirey* (1931).
14. GA Miller, Some effects of intermittent silence. *The Am. journal psychology* **70**, 311–314 (1957).

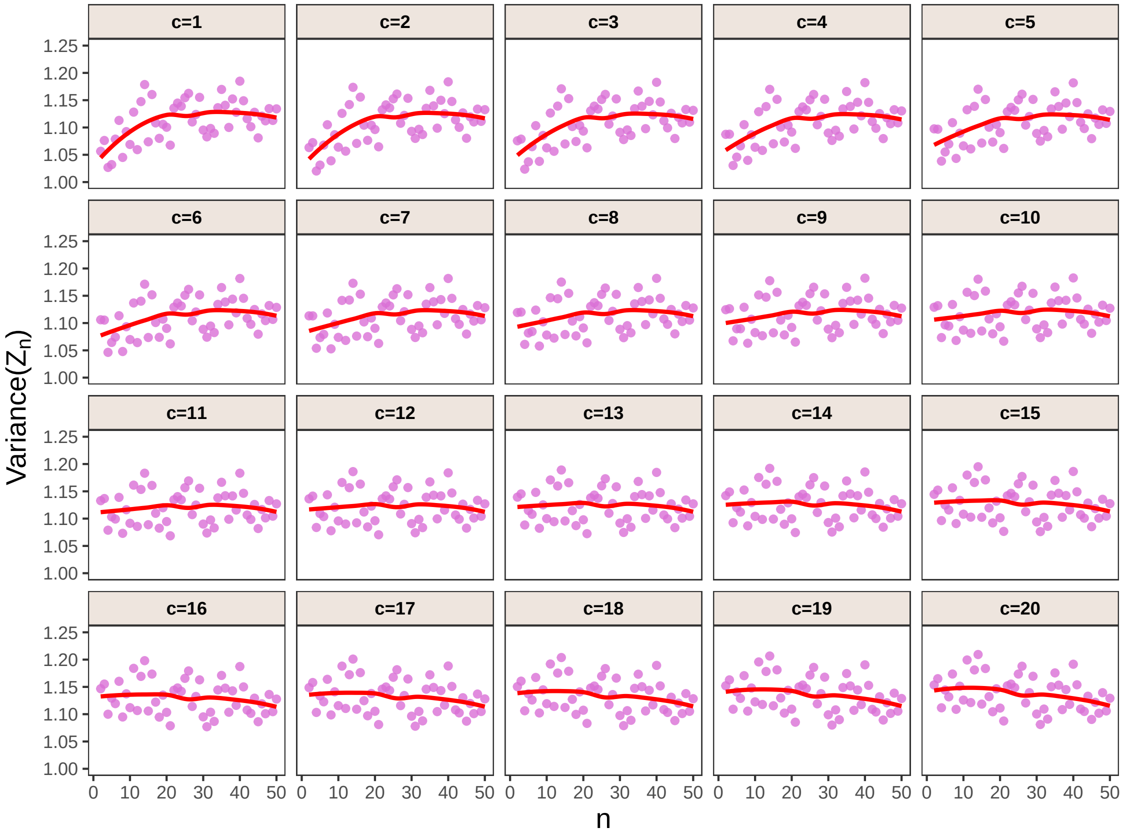

Fig. S3. $Var(Z_n)$ as a function of n for different values of the parameter c.
